# Intonation does aid serial recall after all

**DOI:** 10.3758/s13423-019-01708-4

**Published:** 2020-01-23

**Authors:** Michelina Savino, Bodo Winter, Andrea Bosco, Martine Grice

**Affiliations:** 1 Department of Education, Psychology, Communication, University of Bari, Bari, Italy; 2 Department of English Language & Linguistics, University of Birmingham, Birmingham, UK; 3 IfL-Phonetics, University of Cologne, Cologne, Germany

**Keywords:** Serial recall, Intonation, Working memory, Digit span

## Abstract

A sequence of spoken digits is easier to recall if the digits are grouped into smaller chunks (e.g., through the insertion of pauses). It has been claimed that intonation does not facilitate recall over and above the effect achieved by pauses. This may be related to the fact that past research has used synthesized intonation contours. In this replication study, we show that intonation does provide benefits once more naturalistic intonation contours are used. This benefit is independent of response modality (spoken responses, keyboard responses, or handwritten responses in a grid). We furthermore show that intonation differentially affects specific positions within the sequence of digits. Crucially, our results suggest that researchers and clinicians need to pay attention to intonation when assessing working memory using spoken language.

Serial recall of digits is one of the major ways working memory capacity is assessed, both in research and in clinical settings (Baddeley, Eysenck, & Anderson, 2009; Conway et al., 2005; Wechsler, 1987). It is well established that recall is facilitated when digits are presented in chunks (Crowder & Greene, 2000), especially when presented acoustically (Cowan, Saults, & Brown, 2004; Frankish, 1985), in which the chunks are separated by pauses. What is still unclear is how far intonation—the melody of speech—further facilitates serial recall. According to Frankish (1995) and Saito (1998), intonation can aid recall only insofar as it is involved in chunking, with little effect over and above the effect of pause insertion. In our study, we directly compare the recall of spoken digit sequences grouped by pauses against sequences with specific intonation contours. Our results show that intonation does make a difference after all.

In his study, Frankish (1995) directly compared the effect of intonation and pausing on serial recall using synthesized speech. Pitch contours were obtained by instructing a speaker “to use a strongly-accented intonation pattern to group the sequences into threes” (p. 59); the resulting contours were superimposed onto the synthesized speech. In addition, there was an intonation condition with additional pauses grouping the list into threes, as well as a pause condition with monotone pitch. Frankish found that recall performance did not differ significantly across any of the three grouped conditions (intonation, intonation + pause, pause), which was taken to suggest that pitch movements do not contribute beyond a grouping effect that can be obtained by means of pauses alone.

We reasoned that factors could have hampered the effect of intonation in Frankish’s experiment: (1) The pitch contour may have been unnatural, as it was transplanted from one utterance to another, and (2) the speaker was specifically asked to chunk the list into groups of threes, whereas the intonational structure of lists can have a more complex organization (Hirschberg & Pierrehumbert, 1986; Tyler, 2014). There is cross-linguistic evidence that intonation cues hierarchical grouping in lists, with various rising contours signalling non-final (earlier in lists) and penultimate positions, and falling contours signalling final position (Bolinger, 1989; Geluykens & Swerts, 1994; Ladd, 1980, 2008; Pierrehumbert & Hirschberg, 1990; Savino, 2001, 2004; Savino, Grice, Gili Fivela, & Marotta, 2006; Swerts, Collier, & Terken, 1994). Moreover, when listening, participants were explicitly instructed to use the grouping-into-three strategy and were provided with grids for writing their answers that suggested this grouping. This way of delivering responses biases participants toward chunking, which could have further masked any differences between the different conditions.

In this study, we investigate whether the use of naturalistic intonation patterns conveying positional information facilitates serial recall beyond the grouping effect, especially in certain positions (end of group, end of sequence, penultimate in the group, penultimate in the sequence). To this aim, we replicated Frankish’s (1995) serial recall experiment using natural stimuli with pitch contours conveying positional information. In addition, we compared different recall tasks, including tasks that did not explicitly bias participants toward chunking.

Finally, some studies have reported that recall performance can be facilitated with written-on-grid responses, compared with spoken (Harvey & Beaman, 2007; Penney, 1979) or keyboard responses (Penney & Blackwood, 1989). However, these studies comparing recall modalities did not consider the effect of intonation. Thus, our experiment combines an intonational manipulation (two natural intonation contours, a pause condition, and a no-pause no-intonation control condition) with a response modality manipulation (written-on-grid, keyboard, spoken recall).

## Method

### Conditions

The study was conducted in Italian, specifically the variety spoken in Bari. As is the case with other languages, Bari Italian has a number of pitch contours for cueing positional information of items in sequences (Savino, 2001, 2004; Savino et al., 2006). Positions that are pre-final (penultimate in each triplet and in list) and non-final (any other position that is not final in list) are signalled by different types of rising contours, whereas final position is signalled by a fall (see Table 1 in the Appendix for details on all contour types). On the basis of this tonal inventory, two list types were compiled: Intonation Contour A and Intonation Contour B:

- Intonation Contour A had an intonation contour at the end of the first and second triplets (Positions 3 and 6) signalling non-finality, and a final contour at the end of the entire list (Position 9).
- Intonation Contour B additionally had a contour signalling pre-finality in each triplet, and in list (Positions 2, 5 and 8).

Two additional list types had a neutral falling contour on all digits. The Grouped-by-Pauses condition had a pause after Positions 3 and 6, whereas the Ungrouped condition had no pauses. For a schematic representation of these four prosodic patterns, see Fig. 3 in the Appendix.

### Preparation of stimuli

To construct the stimuli, we first produced sequences of the same digit in all nine positions with Intonation Contour A, Intonation Contour B, and with the neutral falling contour. For example, for digit *uno* (one), the sequence “uno uno uno uno uno uno uno uno uno” was produced once with Contour A, once with Contour B, and once with a neutral falling contour on each digit. In this way, all intonational realizations for each position in each prosodic condition were available for each digit, taking into account downtrends in fundamental frequency (F_0_) across stretches of natural speech (Ladd, 1984). All sequences were produced by a trained speaker of Bari Italian (author M.S.) in the same recording session. All digit renditions were saved as individual audio files and were used as “building blocks” for creating the stimuli for all experimental conditions, by concatenating the individual audio files into nine-digit sequences.

**Fig. 1:**
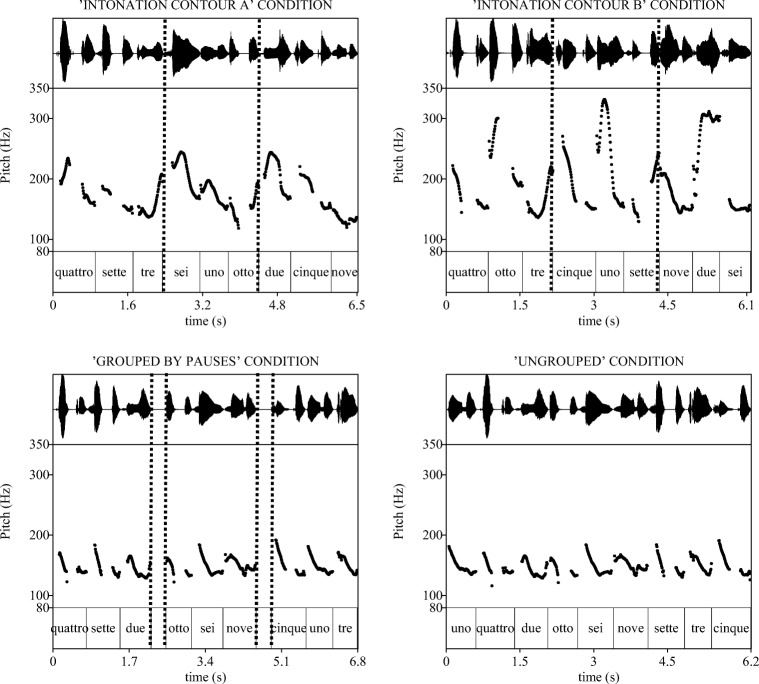
Speech waveform and F_0_ contour of sequence stimuli produced with Intonation Contour A and Intonation Contour B (upper panel), Grouped-by-Pauses and Ungrouped (bottom panel) conditions. In Contour A and B sequences, vertical broken lines mark group boundaries (intonational phrases), whereas in the grouped-by-pause sequence, double vertical broken lines mark silent intervals (pauses) between groups

Spoken digit renditions with the neutral falling pitch shape were concatenated to create sequence stimuli for the conditions Ungrouped (control) and Grouped-by-Pauses. In the latter case, a 310-ms long silence was inserted after digits in Positions 3 and 6. Digits produced with Intonation Contour A and Intonation Contour B were used for creating sequences of these two intonation contour types, respectively. An example of a digit sequence for each of the four prosodic conditions is shown in Fig. 1.

We produced 17 stimuli for each experimental condition, for a total amount of 68, including eight stimuli to be used in the training session (two per prosodic condition). The duration of each stimulus sequence averaged 6.4 s. The concatenated nine-digit sequences were created on the basis of 68 nine-digit lists we derived by pseudo-random permutation of the 1–9 digits, avoiding two adjacent digits in ascending or descending order, or the same digit in an identical position in consecutive lists. All steps for the preparation of stimuli were carried out using Praat (Boersma, 2001).

### Participants

Seventy-eight participants (63 female, 15 male, *M*_age_ = 22.35 years, *SD* = 3.29 years) took part in the experiment for course credit. They were undergraduate and graduate students of psychology at the University of Bari, all born and living in the Bari dialectal area. Participants did not report any speech or hearing deficits, and they did not have any background in phonetics or speech science.

### Procedure

Participants were tested individually in a quiet laboratory, sitting in front of a computer and wearing headphones. They were instructed to listen to each sequence and recall all nine digits in the same order in which they were presented (the importance of recalling in the correct order was emphasized in the instructions). Participants responded immediately after the presentation of the last digit. No grouping strategy was suggested.

Each list was preceded by a 890-ms tone (263 Hz), followed by 500 ms of silence. After each response, participants proceeded to the next sequence by pressing the space bar. They were allowed to pause whenever they wanted during the session, and they were encouraged to take a break after every block of 15 stimuli. Stimuli from the same condition were blocked, with block order balanced across participants. Before starting the task, participants were tested using the WAIS-R Digit Span test (Wechsler, 1987).

In contrast to the stimuli manipulation, which was within participants, the response modality manipulation was between participants. A group of 29 participants (23 female, six male, *M*_age_ = 22.8 years*, SD* = 4.55, digit span = 6.76, *SD* = 0.77) were asked to recall the lists orally. Participants in this condition wore a microphone for recording their responses. Another group of 24 participants (20 female, four male, *M*_age_ = 22.33, *SD* = 2.64, digit span = 6.5, *SD* = 0.96) were instructed to write down each sequence in a nine-box grid drawn on paper, from left to right (in contrast to Frankish, 1995, grids were *not* drawn in a way to overtly suggesting grouping into triplets). They were instructed to fill all nine boxes in the grid even if they were unsure of the correct response. A third group of 25 participants (20 female, five male, *M*_age_ = 21.84, *SD* = 1.46, digit span = 6.48, *SD* = 0.81) performed the task by typing the digits on a computer keyboard, and pressing the “return” key after the end of each recalled sequence. Each session (i.e., including the digit span test, and independently from the recall modality) lasted approximately 40 min. Trials were implemented and run using SuperLab 2.0 (Cedrus Corporation, 1991).

### Statistical analysis

We used R (Version 3.6.0; R Core Team, 2019) and the package brms 2.9.0 (Bürkner, 2017) to compute a mixed Bayesian logistic regression model on the accuracy scores. The main fixed effects were response modality (spoken, keyboard, grid) and condition (Intonation A, Intonation B, pause, control). In addition, we included a fixed effect for “position within triplet,” which was added as a monotonic variable (see Bürkner & Charpentier, 2018). This variable codes for the first, second, and third position within each triplet (1, 2, 3 versus 4, 5, 6 versus 7, 8, 9). Thus, the first “position within triplet” codes for Positions 1, 4, and 7; the second codes for 2, 5, 8; and the third for 3, 6, 9.

As fixed effects, we also included a Position Within Triplet × Condition interaction, as well as a Response Modality × Condition interaction. Digit span and overall position (1 to 9) were added as control variables. To account for primacy and recency effects, we added overall position also as a squared predictor, which models the parabolic shape seen in most serial recall curves. The random effects component included random intercepts for participant as well as random slopes for all within-participant variables (including random slopes for interactions) and correlation terms between all random effect components. Markov Chain Monte Carlo sampling was performed with 4,000 iterations for four chains (2,000 warm-up), resulting in 8,000 posterior samples. There was no indication of any convergence issues (all Rhat = 1.0). Posterior predictive checks indicated no issues.

All data and code for the statistical analyses are made available under the following OSF repository: https://osf.io/5b94c

## Results

**Fig. 2:**
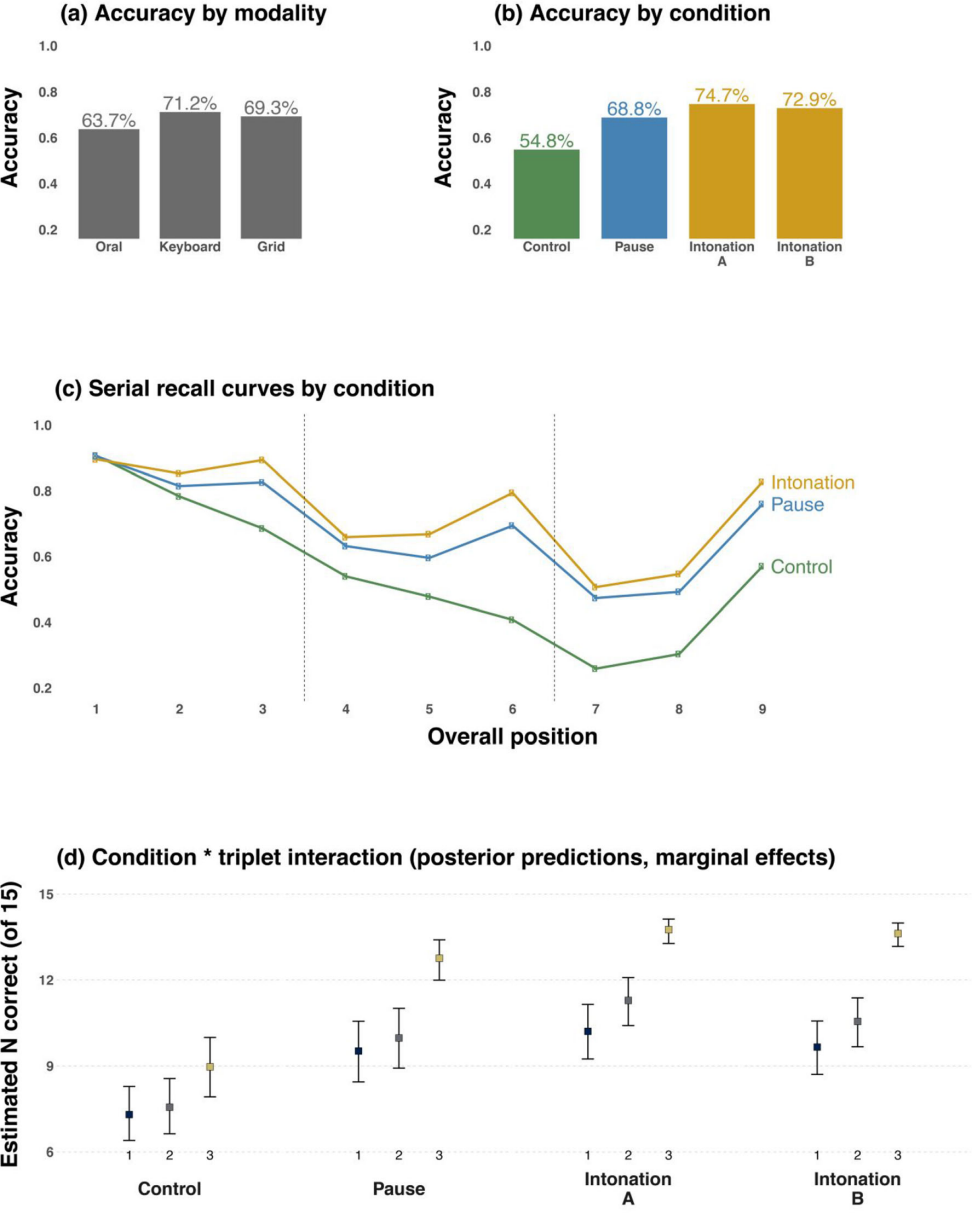
Descriptive accuracy as a function of (**a**) condition and (**b**) modality; (**c**) serial recall curves (aggregated over intonation A & B); (**d**) posterior means and 95% credible intervals for position within triplet (first, second, third), showing that for the intonation conditions, the third position within a triplet was most accurate; this effect was slightly less pronounced for the pause condition, and even less pronounced for the control condition. In the latter condition, an effect appears in the third position of the last triplet only (i.e., the last item in the sequence), as a consequence of the recency effect

**Fig. 3:**
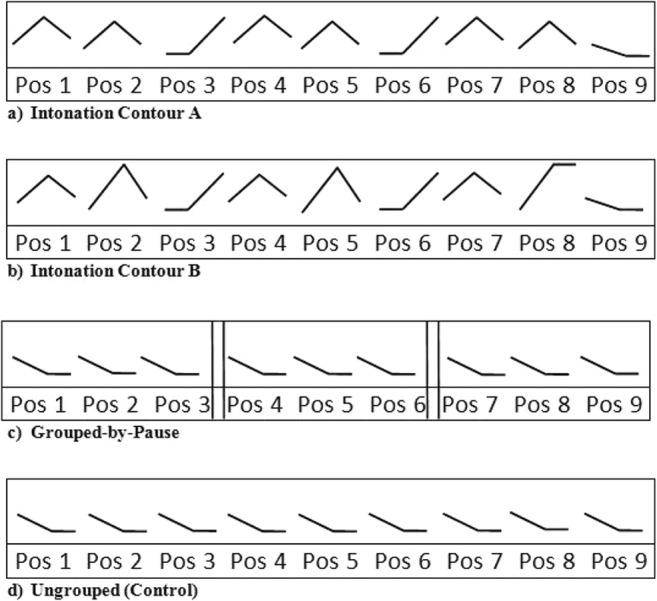
Schematized prosodic patterns of sequence stimuli for each of the four prosodic conditions

Items were scored as correct only if they were recalled in the same serial position in which they were presented. Results of recall performance as a function of output modality show that keyboard responses (71.2%) were on average more accurate than grid responses (69.3%), which were in turn better than spoken responses (63.7%; see Fig. 2a). However, model comparisons (10-fold cross-validation) show that the model with the modality main effect did not lead to better predictions than the model without this effect (*k*-fold IC difference: −4.14, *SE* = 26.98). This suggests that, overall, there are no stark average differences between response modalities in this study.^1^

Overall performance was similar for Intonation Contour A (74.7%) and Intonation Contour B (72.9%), which were both higher in accuracy than the pause condition (68.8%) and the control condition (54.8%; see Fig. 2b). Model comparison with 10-fold cross-validation showed that the model with the condition main effect reliably improved predictive performance (*k*-fold IC difference: 111.35, *SE* = 33.95). In terms of parameter estimates, compared to the control condition (reference level), all three conditions are associated with positive coefficients (higher accuracy) whose 95% Bayesian credible intervals are far from zero: Contour A $\left(\hat{\beta}=1.23, SE=0.11,95\% CI:\left[1.02,1.44\right]\right)$, Contour B $\left(\hat{\beta}=1.06, SE=0.10,\left[0.87,1.25\right]\right)$, and pause $\left(\hat{\beta}=0.85, SE=0.03,\left[0.49,0.61\right]\right)$.^2^ Comparison of the posterior samples for the respective coefficients shows that there is a high posterior probability for contour A (${\hat{\beta}}_A>{\hat{\beta}}_{pause}=1.0$) and contour B (${\hat{\beta}}_B>{\hat{\beta}}_{pause}=0.97$) being overall more accurate than the pause condition. The posterior probability for Contour A being more accurate than B was also high (${\hat{\beta}}_A>{\hat{\beta}}_B=0.98$).

Figure 2c shows the serial recall accuracy curves with the familiar U shape that is generated by primacy and recency effects. The descriptive statistics based on the raw values show that for the first position the average accuracy was quite similar for all non-control conditions (Contour A: 69.8%, Contour B: 67.6%, pause: 67%) when broken up by position within a triplet. However, for the third (final) position within a triplet, both intonation contours, A (83.8%) and B (83.9%), led to much better performances than the pause condition (76.0%). The second (penultimate) position also showed some differences between Intonation Contours A (70.6%) and B (67.2%) compared with the pause condition (63.4%); however, these were not as pronounced. On the other hand, the average accuracy was worse in Contour B than in Contour A for this position, contrary to our expectation. Since in Contour B the penultimate position in a triplet is intonationally marked as such, we expected listeners to make use of that intonational cue, resulting in higher accuracy in recalling that position in B than in A.

Figure 2d shows the marginal posterior predictions of the logistic regression model for position within triplet, broken up by condition. This clearly shows that there was an advantage for the intonation conditions, over and above pause, specifically for the third position within each triplet.

Crucially, the model with the Condition × Position Within Triplet interaction term performed reliably better in terms of predictive accuracy than the model without this term (*k*-fold IC difference: 10946.3, *SE* = 107.8). In terms of parameter estimates, Contours A and B, as well as the pause condition, had interaction coefficients that were far from zero. While the pause condition was associated with a slight boost for later positions within each triplet $\left(\hat{\beta}=0.37, SE=0.05,\left[0.28,0.46\right]\right)$, this boost was much stronger for both contour A $\left(\hat{\beta}=0.60, SE=0.06,\left[0.48,0.72\right]\right)$ and contour B $\left(\hat{\beta}=0.63, SE=0.05,\left[0.52,0.73\right]\right)$. Thus, the intonation contours led to increased accuracy, specifically for later positions within each triplet (see Fig. 2d). A direct comparison of the posterior samples for these interaction coefficients shows that the posterior probability of the interaction with position within triplet was stronger for Contour A than for the pause condition (${\hat{\beta}}_A>{\hat{\beta}}_{pause}=0.99$), and the same was the case for comparing Contour B to the pause condition (${\hat{\beta}}_B>{\hat{\beta}}_{pause}=1.0$). On the other hand, there was little evidence for Contours A and B differing from each other in terms of this interaction effect (${\hat{\beta}}_A>{\hat{\beta}}_B=0.33$). This suggests that both intonation contours receive a similar boost for the position within triplet.

While the position within triplet factor reliably interacted with condition, there was no strong indication for an interaction between response modality and condition. Cross-validation showed that the model with and without this interaction term did not differ reliably in predictive performance (*k*-fold IC = 6.25, *SE* = 24.53).

## Discussion and conclusions

Our results show that when using naturalistic intonation contours, intonation facilitates serial recall as compared with simple pause grouping, especially for items in specific positions: end of first and second triplets, and end of the whole sequence. This indicates that intonation provides an extra cue to chunking, suggesting that a rising intonation marking the end of non-final triplets is perceptually more salient than a pause, highlighting the final digit in the triplet. Similarly, it suggests that a falling intonation at the end of a whole sequence is a clearer signal to finality than a pause.

Our results differ from those obtained by Frankish (1995), possibly for two reasons: (1) In our stimuli, we use a natural (pre-recorded) voice instead of synthesized speech, and (2) unlike English, Italian words for digits are mostly disyllabic, allowing for more time for the intonation contour to unfold, making them better able to lend salience to the digit if a rise is used. Future research needs to replicate this result for other languages to decide between these two explanations; however, regardless of which of these two accounts most likely explains our results, we have shown that intonation does matter after all. Although our results suggest that having many different intonational cues is associated with diminishing returns, as there was no strong difference between Contours A and B, the beneficial effect of intonation in serial recall was strong enough to be consistently observed across all response modalities.

Our findings have implications for methodology in digit span assessment. Current protocol prescribes that clinicians read aloud digit sequences to be recalled by using a “monotone intonation.” Since this is a difficult task, even for trained phoneticians, clinicians are very likely to inadvertently introduce some intonational marking that will differ from one clinician to another. For instance, if an individual is tested at two time points but with a different clinician, a reduction in digit span might be a function of the clinicians’ own intonation patterns rather than a difference in the subject’s working memory capacity. Pre-recorded standardized materials, along the lines of those used in this study (see also Norris, Hall, & Gathercole, 2019, for using a similar methodology in a digit span task), would aid comparability across individuals and sessions. This would be crucial, for example, in longitudinal clinical studies involving digit span assessment.

This paper contributes to our understanding of how human listeners use cues to structure when constructing (and reporting) memory traces. Future work will hopefully contribute toward determining the effects of digit span of stimuli with no pauses, as sometimes occurs in naturally spoken utterances. We hope to inspire further studies on other languages and dialects and how they signal the substructure of grouped lists, and those cue patterns influence digit span.

## Figures and Tables

**Table 1:**
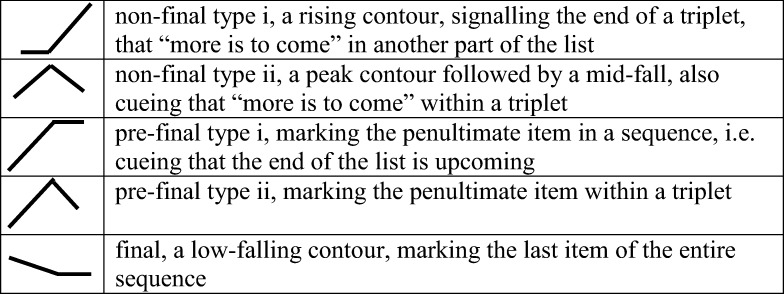
Intonation contour types for signalling item position in lists in Bari Italian

## Footnotes

1 The small differences in performance between response modalities may be related to their intrinsic differences in response time (keyboard responses faster than written on grid, the latter faster than spoken responses due to hesitations or disfluencies). We did not record response time in our study; however, that a different delay in memory recovery (as a consequence of different response time) might influence the recovery process and therefore recall accuracy is a plausible explanation, which is worth verifying in a future study. Thanks to D. Robert Ladd for having brought this issue to our attention.

2 To facilitate the interpretation of these effects, we report coefficients from the model *without* interactions.
